# Investigation of Sheet Metal Forming Using a Rapid Compression Machine

**DOI:** 10.3390/ma12233957

**Published:** 2019-11-29

**Authors:** Sandeep P. Patil, Yann Fenard, Shridhar Bailkeri, Karl Alexander Heufer, Bernd Markert

**Affiliations:** 1 Institute of General Mechanics, RWTH Aachen University, Templergraben 64, 52062 Aachen, Germany; shridhar.bailkeri@rwth-aachen.de (S.B.); markert@iam.rwth-aachen.de (B.M.); 2 Physico-Chemical Fundamentals of Combustion (PCFC), RWTH University, Aachen, Schinkelstrasse 8, 52062 Aachen, Germany; fenard@pcfc.rwth-aachen.de (Y.F.); heufer@pcfc.rwth-aachen.de (K.A.H.)

**Keywords:** rapid compression machine, numerical simulations, sheet metal forming

## Abstract

The primary goal of this work is to understand the deformation behavior of an aluminum alloy (Al) workpiece by using a rapid compression machine (RCM). The primary novelty in this work is that this is the first study on sheet metal forming using RCM. Numerical simulation and experimental results are in excellent agreement, e.g., the dome-shape, the maximum height, the final outer diameter, and the thickness distribution of the deformed workpiece. We demonstrate that the maximum deformation height grows linearly with the peak pressure with an intercept tending to zero. The proposed linear relationship can be effectively used for designing new components for a specific application. Moreover, the proposed numerical model was competent in reproducing the experimental results of damage initiation and evolution in case of high peak pressure as well as the initial misalignment of the workpiece. The results of this investigation revealed that a rapid compression machine can be utilized efficiently for the controlled forming of complex shapes of metal sheets.

## 1. Introduction

The need for manufacturing large and extraordinarily complex parts leads to the development of unconventional techniques. The classic machining of some essential components for the advanced industry limits the development of sectors such as aviation, automotive, defense, or art [1,2,3]. Sheet-metal forming by explosion addresses several crucial points for industrial applications. Explosive forming is applicable to small to large structures, a wide range of materials, complex shapes that are non-accessible by classical machining tools, and it is also cost-effective [1,4]. In this process, an explosion is used as an energy source. One of the cheapest sources of energy is the chemical energy obtained by the fast ignition of a reactive mixture.

To obtain a fast release of chemical energy, a simple way consists of a short-time compression of a reactive mixture to increase its pressure and thus temperature. In conditions of interest, the mixture ignites. At the laboratory scale, it can be achieved in a rapid compression machine (RCM). Typically, RCM is used to measure ignition delay times, an intrinsic combustion parameter of fuel [5,6,7].

In this work, the energy source is a mixture of methane and air. Methane represents an easily and cheaply accessible combustible. The energy release of the mixture is obtained in the RCM at the Physico-Chemical Fundamentals of Combustion (PCFC), RWTH Aachen University. This facility quickly compresses the reactive mixture into a combustion chamber to suitable conditions of pressure and temperature that allow a fast release of the chemical energy as it ignites. The advantages of such a facility are numerous. The filling of the compression chamber with the reactive mixture is fast and economical with fuel thanks to the previously premixed mixture into the vessel prior to experimentation, which can continuously deliver the combustion chamber in the case of several mixing vessels. The combustion chamber, especially the end-plate, can take various shapes, allowing the sheet-metal plate to be formed into various final shapes. The experimental conditions are highly controlled, as the stoichiometry and initial temperature and pressure of the mixture or the stroke and compression ratio, allowing a wide range of pressure release during the ignition that affects the properties of the deformed material. Finally, different gaseous or liquid fuels can be used for the reactive mixture depending on the need.

In the literature, a wide range of experimental investigations and numerical simulation studies are available [8,9,10,11,12]. Moreover, a study of numerical simulations in sheet metal forming and new development possibilities are presented by Tekkaya [13]. However, to the best of our knowledge, this is the first study on the sheet metal forming using RCM. In this work, 54 mm diameter and 1 mm thickness AA 5754 aluminum alloy (Al) workpieces were deformed using experiments. The pressure histories recorded were used to simulate the deformation process of the aluminum sheets numerically. Moreover, in the experiments and simulations, the maximum displacements of the center points of the workpieces are compared and analyzed. Finally, the fully damaged and fractured due to the misalignment of workpieces in the experiments are investigated using the numerical simulations.

## 2. Methods and Setup

### 2.1. Experimental Setup

**Figure 1:**
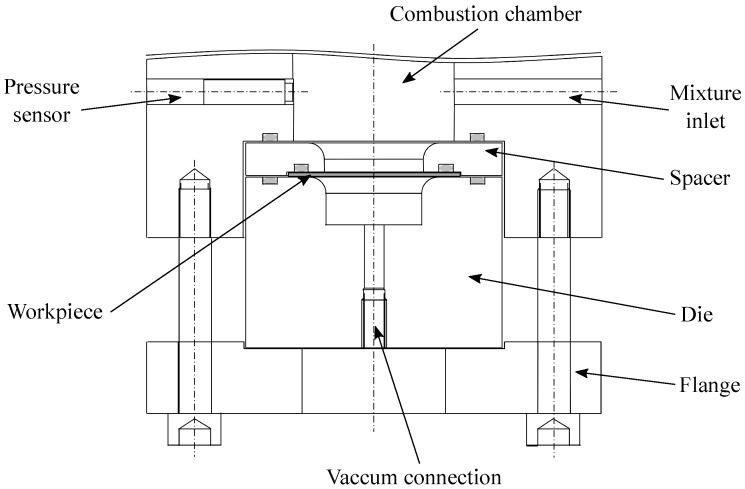
Schematic representation of the experimental setup.

**Figure 2:**
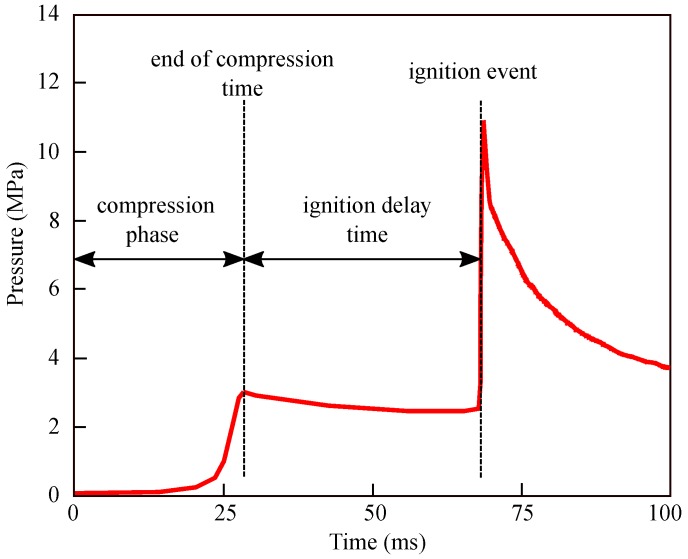
Typical average pressure profile recorded in experiments.

Rapid compression machines have been used since the 1950s to investigate the behavior of the combustion of fuels, especially their ignition delay times (IDTs). Since the early 20th century, rapid compression machines come in different configurations [14]. However, RCMs have in common the stroke of one or two pistons into a combustion chamber to compress a reactive mixture. Figure 1 shows a schematic representation of the experimental setup. It records a pressure history from which the property of the fuel is measured: the ignition delay time. In Figure 2, a typical pressure trace obtained in the PCFC-RCM is illustrated in the case of methane. Three stages are observed. In the compression phase, the mixture goes from initial temperature and pressure to compressed temperature (Tc) and pressure ($p_{c}$). $p_{c}$ and Tc are the parameters controlled and set to be the experimental parameters used to define the experimental conditions. Then, the piston is at its top dead center position, and the pressure slowly decreases due to heat losses, until the rapid increase of the pressure attributed to the homogeneous ignition of the mixture over the entire volume of the chamber. The time elapsed between the pressure $p_{c}$ and the ignition event is the IDT. IDT is defined as the maximum ratio of pressure to time (dP/dt). After the ignition event, because of heat loss, the pressure gradually decreases.

The PCFC-RCM used in this experimental work has been described in detail by Ramalingam et al. [15]. The major details deserving of highlighting are the following. The single-piston is driven by pneumatic air and hydraulically stopped. The reaction chamber consists of a 260 mm long, 50 mm diameter cylinder. Type T thermocouples control the temperature of the gas mixing vessel, manifold, and the reaction chamber. The mixture is prepared by monitoring the partial pressures of gases with suitable range pressure sensors. High purity grade O_2_, Ar (99.999%), and methane (99.95%) were used to prepare the mixture. The experiments were conducted with stoichiometric methane (0.095 %mol)/oxygen (0.190 %mol)/argon (0.715 %mol). The dynamic pressure change in the chamber is recorded with a Kistler 6125C11U20 at 2 MHz frequency with a linear uncertainty of 0.04%.

In the configuration of the present work, the end wall is replaced by dice. Thus, the compression ratio is fixed. Figure 1 describes the geometry of the chamber and the die. The initial pressure was varied to obtain different pressure histories and maximum peak pressures. The maximum peak pressure ranges between 2.45 and 25.1 MPa. This allowed various deformation behavior of the workpiece, where the measured maximal deformation varies from 1.68 to 13.4 mm without failure of the aluminum plate and 15 mm with failure of the plate.

### 2.2. Numerical Modeling

Rapid compression forming is a transient dynamic process where a pressurized gas mixture acts as an energy source and impinges on the Al workpiece to be deformed. As this process involves short-time dynamics, explicit schemes are well suited to describe the deformation. Hence, the solution of dynamic equilibrium equations forms the basis of the simulation [16]. Explicit time integration finite element (FE) solver LS-DYNA is used to perform all the simulations (version: ls971 R7.1.1) [17]. As the deformation of the workpiece is of primary interest and not the pressure wave propagation, the FE model is simplified by direct application of pressure profile obtained from the experimental recording as a load on the Al sheet in all the simulations.

**Figure 3:**
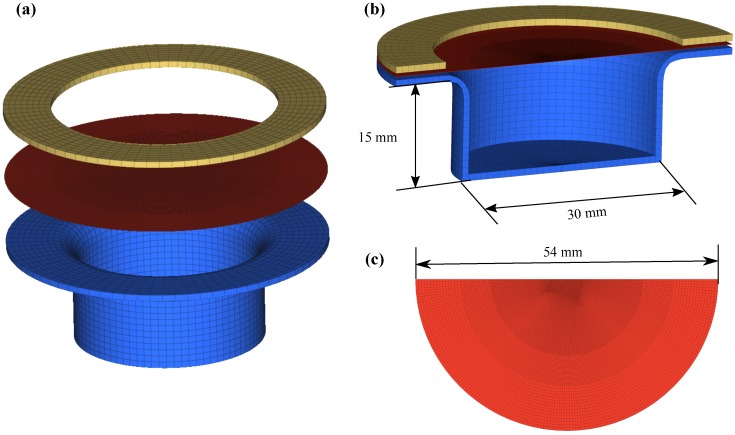
(**a**) Schematic representation of the full 3D finite element (FE) model. The die (blue) and spacer (yellow) are constructed with the solid elements. Half-sectional view of (**b**) the full model and (**c**) the meshed workpiece of the shell elements.

In this work, the full 3D models were constructed to understand the deformation mechanism of the workpiece with miss-alignment (offset) and the location of damage initiation and nature of its evolution. Figure 3 describes the FE model details. The die and holder plate (at the top of the image in Figure 3, also known as the spacer) were assigned as the rigid materials (MAT_020) because they possess high stiffness when compared to the sheet to be formed, and during the forming process, these components were in-active. The Belytschko–Tsay [17] shell element formulation was chosen for the forming sheet. In the present work, the mesh refinement is obtained by h-refinement. As the number of degrees of freedom increase (very coarse to very fine mesh), internal energy also increases, and after fine mesh, it shows nearly stable values. Therefore, the workpiece consists of a total of 50,560 elements. For more details, the reader is referred to Patil et al. [11]. The die and top plate were fixed, and segment based surface-to-surface contact formulation (penalty method) [17] was used between each of them and the forming sheet, assuming planer segments. The free surface of the forming sheet was used for the application of pressure. For more details of modeling and simulations, the reader is referred to Patil et al. [11,12]. The mechanical properties of the aluminum sheet are given in Table 1.

In the literature, the Johnson–Cook material model (MAT_015) [19] possesses the potential to demonstrate material responses, such as high-speed forming due to impact load. It includes strain hardening, strain rate sensitivity and thermal softening responses of the material. These responses are combined in a multiplicative manner. Therefore, in this work, this material model is used to simulate the workpiece deformation in the RCM process. In our previous work [9,10,11,12], the Johnson–Cook material model was used for gas detonation forming simulations, wherein simulation results were in good agreement with experiments. Johnson–Cook constitutive stress reads as

(1) $$\sigma_{y}=\left(A+B{\bar{\varepsilon }}^{p^{n}}\right)\left(1+C\ln\frac{{\dot{\bar{\varepsilon }}}^{p}}{{\dot{\varepsilon }}_{0}}\right)\left(1-{\left[\frac{T-T_{room}}{T_{melt}-T_{room}}\right]}^{m}\right)\;$$

where ${\bar{\varepsilon }}^{p}$ is the effective plastic strain, which is calculated incrementally using the equivalent plastic strain rate, and it increases whenever the material is actively yielding. $T_{room}$ is the ambient temperature, $T_{melt}$ is the melting point or solidus temperature, *T* is the effective temperature, *A* is the yield stress, *B* is the hardening modulus, *n* is the strain exponent, *m* is the temperature exponent, and *C* is the strain rate factor. Furthermore, ${\dot{\varepsilon }}_{0}$ is the quasi-static threshold strain rate ${\dot{\varepsilon }}_{0}=1.0\times 10^{-5}\;{\mathrm{ms}}^{-1}$ [17].

The term in the first bracket describes isothermal static material behavior on the right-hand side of Equation (1), i.e., the strain hardening of the yield stress. Static tensile tests determine the parameters *A*, *B*, and *n*. Strain rate hardening parameter *C* is represented by the second term. Local thermal effects cause softening of the yield stress and the last term represents it. In this work, the thermal softening effect was not considered. Material parameters of the simulations are given in Table 2.

Void nucleation, growth, and coalescence define the three phases of rupture in the materials [23,24]. Equivalent plastic strain, triaxiality (ratio of the mean stress to the von Mises effective stress), load type, and geometry affect the damage behavior of the materials. Moreover, the strain rate influences the damage behavior. Failure strain is presented by

(2) $$\varepsilon^{f}=\;\left[D_{1}+D_{2}\exp\left(D_{3}\sigma^{*}\right)\right]\left[1+D_{4}\ln{\dot{\varepsilon }}^{*}\right]\left[1+D_{5}T^{*}\right]\;$$

where $D_{1}$ to $D_{5}$ are constants. $\sigma^{*}$ is the ratio of pressure divided by effective stress

(3) $$\sigma^{*}=\frac{P}{\sigma_{eff}}\;$$

where *P* is the average of the normal stresses and $\sigma_{eff}$ is the von Mises equivalent stress. ${\dot{\varepsilon }}^{*}$ is the normalized effective plastic strain, given by

(4) $${\dot{\varepsilon }}^{*}=\frac{{\dot{\bar{\varepsilon }}}^{p}}{{\dot{\varepsilon }}_{0}}.$$

The expression in the first set of brackets in Equation (2) represents that the strain to fracture decreases as the average normal stresses, *P*, increases. The second set of brackets represents the effect of the strain rate and the third set of brackets represents the effect of temperature [20]. Zhao and Kong [25] proposed that the $D_{1}$ parameter is sufficient to describe the damage in the aluminum alloy in the case of high-speed impact (ultra-high pressure of projectile impacting target plate). The proposed value was 0.8 for $D_{1}$ parameter. The damage to an element is defined as

(5) $$D=\sum \frac{\Delta {\bar{\varepsilon }}^{p}}{\varepsilon^{f}}\;$$

where $\Delta {\bar{\varepsilon }}^{p}$ is the increment of the equivalent plastic strain, which would occur during the integration cycle, and $\varepsilon^{f}$ is the equivalent strain to fracture under the current condition of pressure, equivalent stress, and strain rate. A fracture occurs when the damage parameter *D* reaches the value of 1 and delete the corresponding failed elements.

## 3. Results and Discussion

### 3.1. Dome Height and Outer Diameter

A number of iterations were carried out on a rapid compression machine for an aluminum sheet of thickness 1 mm and diameter 54 mm. A wide range of peak pressure values from 2.45 to 25.1 MPa was tested. This generated high-pressure impacts on the Al sheet. The workpiece is axially inline, which is in between the holder and the die. In the previous work, Patil et al. [9] and Yasar [8] investigated that if the applied load is triangular, one can see the spring-back effects. Therefore, actual pressure profiles recorded from the experiments are crucial, and one has to consider these profiles for the numerical simulations. In this work, numerical simulations were performed using the recorded experimental pressure profile, which was acted as a load on the workpiece. In experiments and simulations, the spring-back effect, as well as wrinkles on the flange or the bend region, were not observed.

**Figure 4:**
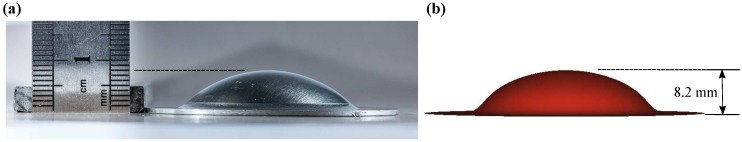
(**a**) Formed cup in the rapid compression machine (RCM). (**b**) Cup formation predicted by numerical simulations.

**Figure 5:**
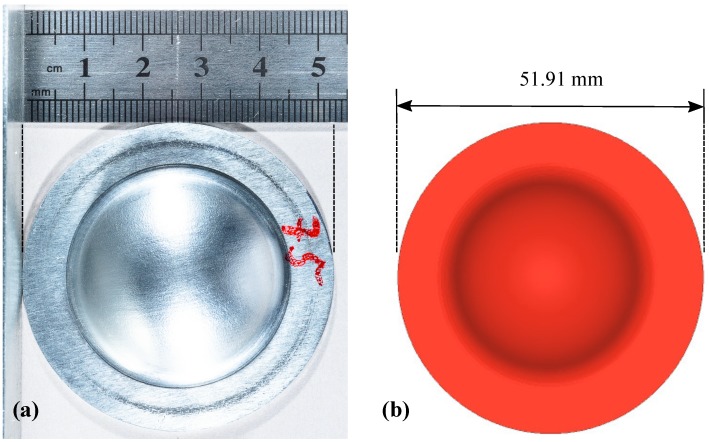
Comparison of diameters of (**a**) experimentally- and (**b**) numerically-formed cups.

A comparison of the deformed shapes in the experiment and the numerical simulation is shown in Figure 4. The observed dome heights were 8.0 mm and 8.2 mm in experiment and simulation, respectively. Moreover, for this particular case of 12.8 MPa peak pressure, the numerical simulations showed a qualitatively similar dome-shape as that of the experiments. However, one can observe the minimal differences in the curvature of the dome. This might be due to a uniform-pressure load in numerical simulations and a slight variation in uniform-pressure in experiments. Final diameter comparison in the experiment and simulation is shown in Figure 5. The obtained final diameter in the numerical simulations was 51.91 mm, which was in good agreement with the experimental value of 52.70 mm.

Initially, static and dynamic coefficients of friction of 0.12 were considered between the workpiece and the top plate, and 0.08 was considered between the workpiece and die [26]. However, the outer diameter was nearly 53.5 mm. In the case where the friction parameters were zeros, the outer diameter nearly matched to the experimental diameter 52.70 mm. Therefore, in this work, numerical simulations were performed using the friction parameters of zeros.

**Figure 6:**
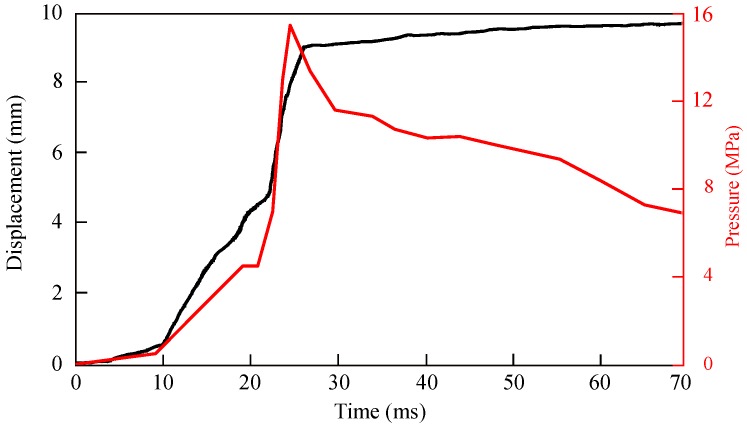
Displacement of the center point during cup formation and pressure profile input in simulation.

In the numerical simulation, the center point displacement of the cup and pressure load magnitude applied on the cup with respect to time is shown in Figure 6. It is observed that initially, there is no significant displacement of the center point due to only compression pressure (no ignition). However, at the instantaneous pressure rise, there was a high increase in the displacement. The peak pressure observed at ∼25 ms. After the peak point, the pressure decreases significantly. Therefore, the displacement increased substantially up to ∼27 ms, which was after the peak pressure time. Subsequently, one can observe a steady increase in displacement. Similar observations were made in our previous work [9,11,12]. In summary, the rate of increase of pressure, as well as the peak pressure value, influences the nature of the increase of the displacement.

**Figure 7:**
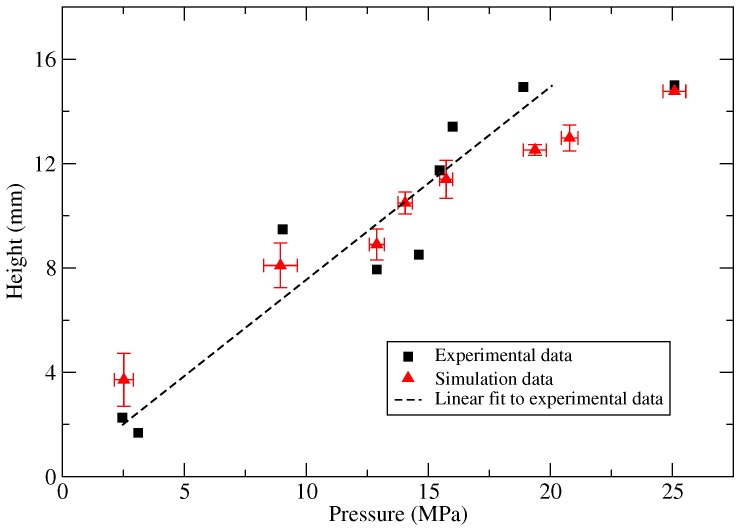
Comparison of dome heights from a series of experiments and numerical simulations. Results of FE simulations with standard error bars.

The pressure applied to the workpiece implies its deformation into the die. To investigate the influence of the peak pressure to the maximum deformation, the maximum height experimentally, as well as numerically observed for each shot is plotted against the respective peak pressure in Figure 7. Here, the maximum height is defined as the displacement of the center point during cup formation. The experimental data show a global linear correlation between the peak pressure and the maximum deformation with an intercept tending to null.

The maximum height, 15 mm, was observed for pressure greater than or equal to 18 MPa, keeping in mind that it is the depth of the die. However, the maximal displacement of 15 mm was not observed without any damage to the workpiece. It is also observed that the experimental data points were more scattered. The maximal standard deviation observed is 2 mm for the maximal displacement. Moreover, the peak pressure represents a source of uncertainty. The pressure sensor used in experiments exhibits very low uncertainty on the pressure measure (0.04%). However, the peak pressure event is an extremely short event and represents a sharped trend. The frequency of recording (2 MHz) is kept higher than the natural frequency (70 KHz) of the sensor, which is a limitation. Due to this limitation, the actual peak pressure can be missing by the recording process, and printed peak pressure can be smoothed. As a consequence, we can estimate an uncertainty on the peak pressure of 1 MPa, which could explain the scatter shown in Figure 7. Another influencing parameter is the slope of the peak pressure during the ignition of the mixture. This is strongly impacted by the combustion behavior of the fuel, and the physical conditions of the experiment, such as the amount of fuel in the mixture, the equivalence ratio, the compressed temperature and pressure. In this work, only the compressed pressure and temperatures are varied. In the numerical simulations, the variation of the peak pressure was considered and obtained heights were plotted in Figure 7.

#### Thickness Variation

**Figure 8:**
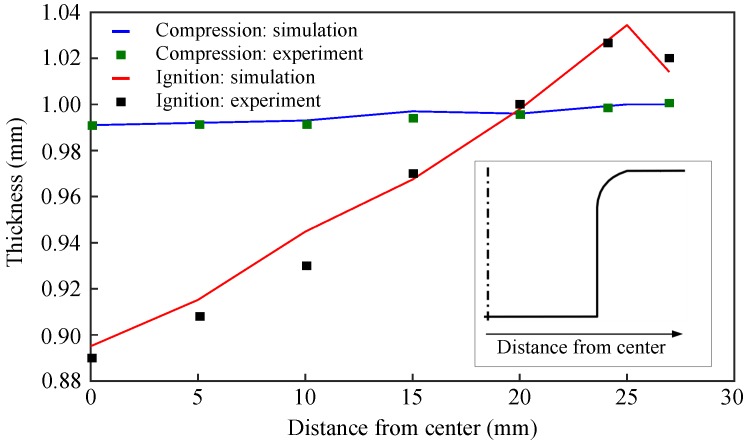
Reported thickness variation of the formed workpiece for no ignition and ignition of reactive mixture. Inset: Section view of the die.

Figure 8 describes the thickness distribution in the deformed workpiece with respect to the initial radius of the die. In our previous work [9,10,11,12], it was concluded that the pressure loading rate (slope of pressure profile) affects the thickness distribution. Hence, it becomes important to have an accurate loading curve from the experiment. It is a well-known fact in the forming process that the method with which the forming sheet is held between the holder and the die influences significantly the deformation behavior. For example, the rigidly clamped sheet results in the maximum thickness reduction. On the contrary, allowing the sheet to slide horizontally between the holder and die results in the least thickness reduction, hence the latter case is a preferred one.

In this work, predicting the thickness distribution accurately of the deformed cup was one of the objectives of the numerical simulation studies. Along the initial radius of the workpiece, the thickness distribution predicted by numerical simulations is shown in Figure 8. Simulations have closely captured the thickness distribution that occurred in the experiments. At the bend region of the die, 1 % thickness reduction of the deformed sheet was observed for the ignition, as well as, compression cases. Moreover, at the center of the die, nearly 11% and 1% thickness reductions were noticed for the ignition and compression without ignition cases, respectively. This stretching of the sheet can be attributed to its hard contact with the die and the holder. However, nearly 3% thickness increase was observed in the flange region, which was the consequence of the tangential compression stresses in that region of the flange being dominant against the tensile meridional stresses.

### 3.2. Damage in the Workpiece

The nature of failure caused by damage evolution is an essential factor in the correlation between the experiments and simulations. The fidelity of simulation results is based on how well the FE model replicates the results of experiments. The damage analysis can provide us the failure patterns as well as an accurate idea of pressure magnitudes, which can cause the failure of the workpiece. In this work, fracture initiation was defined by tensile failure stress. Shell elements are deleted when stress occurred in the model equals or greater than tensile failure stress [18].

**Figure 9:**
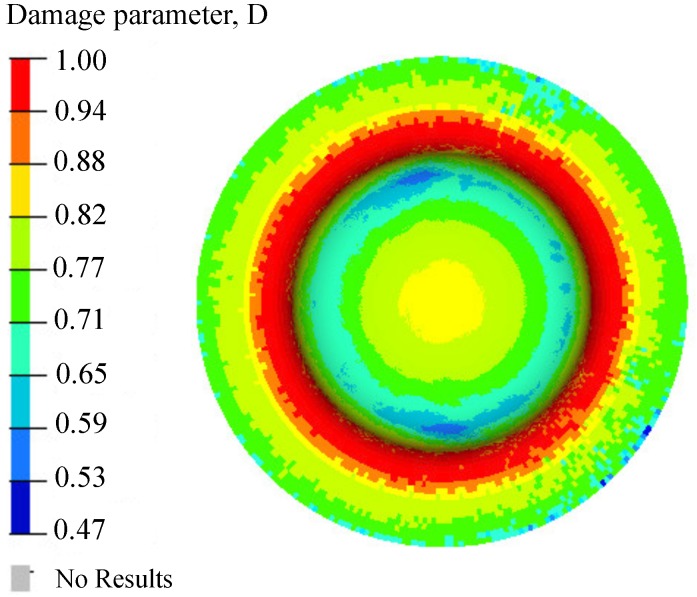
The snapshot of distribution of damage parameter *D* in the case of high-pressure loading (just before D=1).

**Figure 10:**
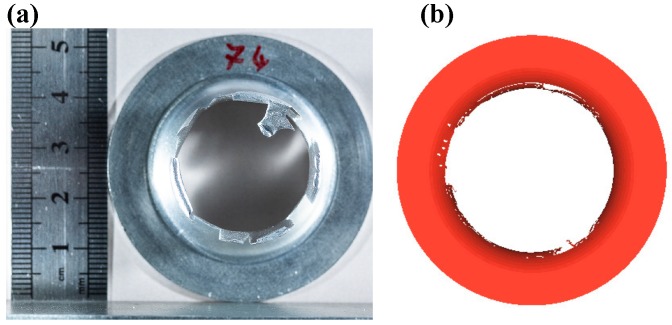
Snapshots of the fully damaged samples: (**a**) experiment and (**b**) numerical simulation.

The damage initiation was seen with the case of peak pressure of magnitude 25 MPa. Figure 9 shows the damage parameter distribution. The critical region was the fillet one, wherein the workpiece started bending to form a cup. Hence, the stress hot spot can be observed as a circumferential band in this region, and finally, the first point of damage initiation was in this circumferential banded region. Figure 10 depicts the damaged samples in experiment and simulation, which shows remarkable similarities in nature of failure. Many parameters play an important role in damage initiation and propagation, for example, the shape of the die, nature of loading, material defects, sheet thickness, initial voids, and impurities present in the workpiece. Therefore, in our previous work [11], the damage initiation of DC04 steel was observed at the bottom of the die.

### 3.3. Effect of Misalignment

**Figure 11:**
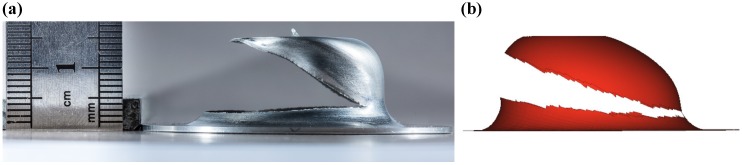
Damage caused by the misalignment of the workpiece: (**a**) experiment and (**b**) numerical simulation.

In the experimental setup, there were possibilities that the workpiece was not perfectly aligned with respect to the die, resulting in a misalignment (offset). A couple of damaged samples were observed in the experiments. Therefore, simulations were carried out with the workpiece having a 2 mm offset with respect to the center of the die, which was nearly the same as that of the experiments. Due to the offset, the contact area between the workpiece and the die at the flange region was changed. The flange region has a larger area on one side and the smallest on the diametrically opposite side. As loading continues on the workpiece, the model reaches the failure stress, and element deletion occurs, which results in failure at the flange area of less-contact. The failure patterns (open-up) of the model as shown in Figure 11. In the experiments and numerical simulations, the maximum displacement of 15 mm was achieved due to the limit of die-depth. Moreover, the outer diameters were nearly the same. Therefore, the experiment and simulation show excellent agreement.

## 4. Conclusions and Future Work

This work proved that a rapid compression machine had demonstrated the capability to deform the aluminum workpiece into the dome-shaped cup without any observable wrinkles. Numerical simulations were performed using the Johnson–Cook material model.

The numerical method and experimental results are in excellent agreement, e.g., the dome-shape, the maximum height, the final outer diameter, and the thickness distribution of the deformed workpiece. The experimental data show a global linear correlation between the peak pressure and the maximum deformation height with an intercept tending to null. The proposed linear relationship can be effectively used for designing new components for a specific application. Moreover, the proposed numerical model was competent in reproducing the experimental results of damage initiation and evolution in case of high peak pressure, as well as, the initial misalignment of the workpiece.

The future outlook of this work can be made by performing a number of experiments and numerical simulations of a wide range of complex geometries. In the numerical study, temperature effects can be included and compared to the experiments. Moreover, the slope of the pressure profile during the ignition process can be investigated in detail to understand its effect on the deformation behavior of a workpiece. Furthermore, this investigation could be coupled with a kinetic modeling tool to achieve refinement of reactive mixture properties to obtain the desired shape and deformation properties of the material.

## Figures and Tables

**Table 1:** Mechanical properties of Al [18].

Young’s modulus (GPa)	68.9
Poisson’s ratio	0.3
Density (kg/m^3^)	2770
Tensile strength (MPa)	353

**Table 2:** The parameters of the Johnson–Cook material model [20,21,22].

Yield stress, *A* (MPa)	67.4
Strength coefficient, *B* (MPa)	471
Deformation hardening, *n*	0.424
Strain rate coefficient, *C*	0.003
Deformation sensitivity, *m*	2.52
